# Improving Thermal Conductivity of Injection Molded Polycarbonate/Boron Nitride Composites by Incorporating Spherical Alumina Particles: The Influence of Alumina Particle Size

**DOI:** 10.3390/polym14173477

**Published:** 2022-08-25

**Authors:** Chuxiang Zhou, Yang Bai, Huawei Zou, Shengtai Zhou

**Affiliations:** State Key Laboratory of Polymer Materials Engineering, Polymer Research Institute, Sichuan University, Chengdu 610065, China

**Keywords:** injection molding, polycarbonate, boron nitride, spherical alumina, thermal conductivity, microstructure, mechanical properties, electrical insulation

## Abstract

In this work, the influences of alumina (Al_2_O_3_) particle size and loading concentration on the properties of injection molded polycarbonate (PC)/boron nitride (BN)/Al_2_O_3_ composites were systematically studied. Results indicated that both in-plane and through-plane thermal conductivity of the ternary composites were significantly improved with the addition of spherical Al_2_O_3_ particles. In addition, the thermal conductivity of polymer composites increased significantly with increasing Al_2_O_3_ concentration and particle size, which were related to the following factors: (1) the presence of spherical Al_2_O_3_ particles altered the orientation state of flaky BN fillers that were in close proximity to Al_2_O_3_ particles (as confirmed by SEM observations and XRD analysis), which was believed crucial to improving the through-plane thermal conductivity of injection molded samples; (2) the presence of Al_2_O_3_ particles increased the filler packing density by bridging the uniformly distributed BN fillers within PC substrate, thereby leading to a significant enhancement of thermal conductivity. The in-plane and through-plane thermal conductivity of PC/50 μm-Al_2_O_3_ 40 wt%/BN 20 wt% composites reached as high as 2.95 and 1.78 W/mK, which were 1183% and 710% higher than those of pure PC, respectively. The prepared polymer composites exhibited reasonable mechanical performance, and excellent electrical insulation properties and processability, which showed potential applications in advanced engineering fields that require both thermal conduction and electrical insulation properties.

## 1. Introduction

Nowadays, the booming development in the fields of new energy sectors, cloud computing, the Internet of Things, high-speed communication, and artificial intelligence are imperceptibly changing our daily life. However, heat dissipation is a growing concern due to the integration of multifunctional components in confined areas, especially in the fields of microelectronics [1], battery units [2], and new energy sectors [3]. Thermally conductive polymer composites demonstrate an edge over metals and ceramic materials in terms of weight advantage, excellent resistance to corrosive environments, and most importantly, good processability and moldability [4,5], which can be scaled at large quantities without causing much additional costs [6]. Therefore, developing high performance thermally conductive polymer composites has become the core interest of researchers from both academic and industrial spheres.

It is known that the intrinsic thermal conductivity of polymers is very low (0.1–0.5 W/mK), which cannot meet the stringent requirements of industrial sectors [2,7]. Thus, a great effort has been devoted to developing highly thermally conductive polymers to suit the needs of the above-mentioned fields. The synthesis of intrinsically thermally conductive polymers is currently impractical, since very complex processes are involved, and the yields are always not satisfying [8,9,10]. In addition, synthesizing intrinsically thermally conductive polymers is costly, and only a few types of materials are available that are not cost effective and also not compatible for large scale industrial applications [11,12]. The commonly accepted approach is adding thermally conductive fillers to the polymer matrix using either solution [13,14] or melt blending methods [15,16,17]. Although solution mixing achieves better filler distribution and the prepared composites exhibit higher thermally conductive properties [18], the use of large amounts of solvents limits its wide use in industrial sectors [19]. Therefore, the melt blending method, which is industrially compatible and environmentally benign, has been widely adopted to prepare thermally conductive polymer composites [20,21].

Conventionally, different types of thermally conductive fillers such as carbonaceous fillers, metallic fillers, and ceramic fillers are adopted for preparing thermally conductive polymer composites [22]. However, the use of metallic [23] and carbonaceous [24,25] fillers impairs electrical insulation properties of polymer composites, which restricts their applications in fields that require electrical insulation performance. As a result, ceramic fillers such as boron nitride (BN) [26], alumina (Al_2_O_3_) [27], and aluminum nitride (AlN) [2] are commonly employed to prepare thermally conductive yet electrically insulative polymer composites. It has been accepted that the formation of a thermally conductive network and the increase of filler packing density are prerequisites for achieving thermally conductive polymer composites [28]. Under such circumstances, filler concentrations as high as 30 vol% are not rare in terms of preparing thermally conductive polymer composites [2]. Thus, great effort has been paid to constructing thermally conductive pathways and increasing filler packing density while not significantly impairing the mechanical and processing properties.

BN, which exhibits a planar structure alike flake graphite has been widely adopted to prepare thermally conductive polymer composites due to its intrinsically high thermal conductivity and thermal stability [29,30]. Injection molding, which is geared towards mass production at industrial scales, exerts a complex shearing influence on polymer melts that leads to a preferential orientation of planar fillers in injection molded articles [31,32,33]. In this scenario, a great discrepancy was noted in terms of the measured thermal conductivity with respect to melt flow direction, i.e., in-plane (along the flow direction) and through-plane (perpendicular to the flow direction) directions [34,35]. For example, there is a great possibility of forming intact filler conductive pathways along the melt flow direction due to the preferred orientation of planar fillers, whereas the properties in pathways perpendicular to the flow direction are significantly impaired [36,37]. As a result, there is a great anisotropy in the values of thermal conductivity for injection molded samples related to the melt flow direction [38].

Presently, numerous studies have indicated that hybrid filler loading is effective in improving the thermal conductivity of polymer composites by facial construction of thermally conductive pathways [39,40,41]. In a previous study [42], we found that the incorporation of spherical Al_2_O_3_ particles was effective in altering the orientation state of planar BN fillers in polycarbonate (PC)-based composites, thereby minimizing the difference between in-plane and through-plane thermal conductivity. In addition, Liu et al. [43] reported that the addition of a small amount (5 wt%) of spherical graphite was instrumental in building a more compact and denser filler packing structure in 45 wt% flake graphite (FG)-filled polypropylene (PP) composites, which was beneficial to improving the thermal conductivity of the resultant moldings. In another work [44], the same authors reported that the loading of spherical Al_2_O_3_ particles was able to affect the orientation state of FG in PP-based composites. As a result, both in-plane and through-plane thermal conductivity were enhanced for PP/FG composites. Moreover, they reported that the size of Al_2_O_3_ particles played a role in building thermally conductive pathways, and the addition of smaller size Al_2_O_3_ particles was proven to be more effective. However, the above studies were performed using the compression molding method, which exerts much lower shearing impact on the polymer melts when compared with the injection molding process [45]. To the best of our knowledge, the influence of spherical particle size on the distribution state of planar fillers under the influence of high shear rates has scarcely been studied.

To attempt to bridge this knowledge gap, spherical Al_2_O_3_ particles of different sizes were employed by using PC/BN composites as the model systems, and the properties such as thermal conductivity, morphological and mechanical properties, as well as rheological properties of ternary PC/Al_2_O_3_/BN composites were systematically investigated. In this work, we reported that both the loading content and the size of spherical particles played a role in determining the distribution state of planar BN fillers. This work provided a new perspective in simultaneously improving the in-plane and through-plane thermal conductivities of injection molded polymer composites without significantly impairing the processability and mechanical properties, which show potential applications in the fields that require both thermal dissipation and excellent electrical insulation properties.

## 2. Experimental Section

### 2.1. Materials

Polycarbonate (PC, L-1225M) with a melt flow index of 28.8 g/10 min (300 °C @ 1.2 kg load) was produced by Teijin Polycarbonate China Ltd. (Jiaxing, China). Two-dimensional boron nitride (BN) fillers with an average size of 35 μm were purchased from Dandong Rijin Science and Technology Co., Ltd. (Dandong, China). Spherical alumina (Al_2_O_3_) with respective particle sizes of 5 and 50 μm were provided by Zhengzhou Sanhe New Materials Co., Ltd. (Zhengzhou, China).

### 2.2. Preparation of Samples

Briefly, PC, BN, and Al_2_O_3_ were thoroughly dried at 60 °C for at least 10 h prior to melt blending. Then a series of filler-containing PC-based composites (Table 1) was prepared using a twin-screw extruder (TSSJ-25/33, Chengdu Tarise Chemical Engineering Co. Ltd., Chengdu, China). The screw rotation speed was set at 30 rpm. The temperatures from the hopper to die zones were set from 240 to 260 °C. Then, the extrudates were pelletized, fully dried under the above-mentioned conditions and used for injection molding using an MA-2000 injection molding machine (Ningbo Haitian Machinery Inc., Ningbo, China). The melting and mold temperatures were set at 280 and 100 °C, respectively. The injection speed was set at 150 mm/s. The approximated shear rates were higher than 10^5^ 1/s. The larger size, i.e., 50 μm, Al_2_O_3_ particles were denoted as A_L_, the smaller Al_2_O_3_ particles were named as A_S_, and the BN was abbreviated as B. Thus, PC/50 μm-Al_2_O_3_ 40 wt%/BN 20 wt% composites were termed as PC/A_L_40B20. The same nomenclature system was applicable to the other systems, as listed in Table 1.

### 2.3. Characterization

The thermal conductivity (λ) of PC-based composites was measured using a LFA467 flash apparatus (NETZSCH, Selb, Germany). Samples with a diameter of 25 and a thickness of 0.2 mm were adopted. It should be noted that this technique reports λ in both the in-plane and through-plane directions. Five replicates were tested for each sample.

The morphology of filler-containing composites was observed by a scanning electronic microscope (SEM; Thermoscientific Apreo S, Oxford Instruments, Abingdon, UK). All samples were fractured in liquid nitrogen, and then the cryo-fractured samples were coated with gold to enhance image resolution.

XRD patterns of the composites were collected using an X-ray diffractometer (Ultima IV, Rigaku, Tokyo, Japan), and the scans were conducted in a 2θ range of 20–60° at a scanning speed of 2°/min.

The viscoelastic properties of samples with a diameter of 25 mm and a thickness of 1 mm were determined using a dynamic rheometer (MCR302, Anton Paar, Graz, Austria). The test was carried out under a constant-strain mode, where the applied strain was set at 1%. The scan frequency ranged from 100 to 0.01 Hz, and the test temperature was set at 260 °C.

The tensile strength was determined using a UTM4204 universal tester (Shenzhen Suns Company, Shenzhen, China) at 10 mm/min as per GB/T 1040.2-2006. The bending tests were carried out on a UTM4204 universal tester according to GB/T 9341-2008. The impact strength of specimen with a 2 mm V-notch was measured in accordance with GB/T 1843-2008. Five replicates were tested for each sample.

The resistance (R_x_) of samples was measured using a high resistance meter (ZC-90F, Shanghai Taiou Electronics Co., Ltd., Shanghai, China), and the volume resistance (ρ_v_) was obtained using the following equation:

(1) $$\rho_{v}=R_{x}S/L$$

where *L* is the distance between the electrodes, and *S* is the cross-sectional area of the samples. Three replicates were tested for each sample.

## 3. Results and Discussion

### 3.1. Thermal Conductivity of PC/Al_2_O_3_ Composites

**Figure 1:**
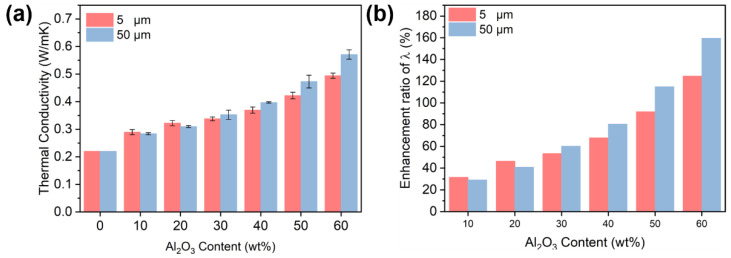
(**a**) Thermal conductivity (λ) and (**b**) enhancement ratio of λ of PC/Al_2_O_3_ composites in comparison with pure PC: influence of particle size and filler concentration.

**Figure 2:**
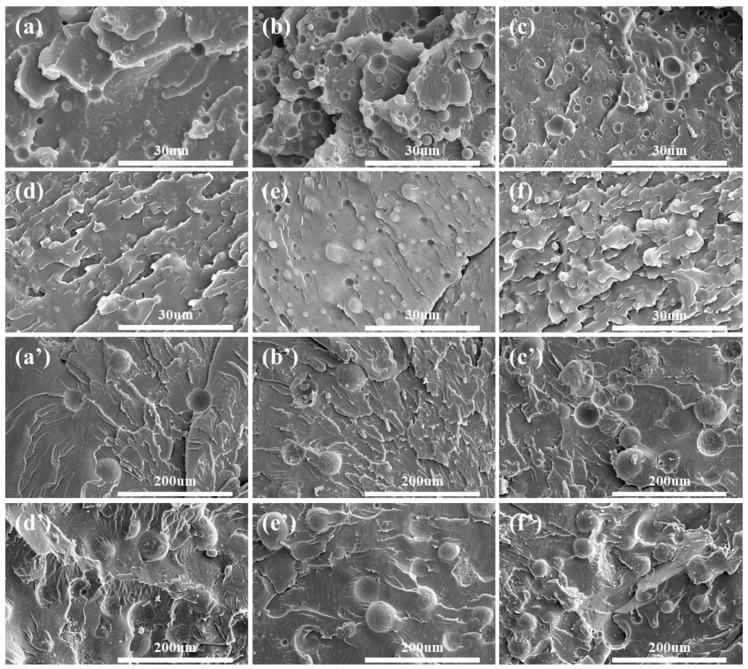
SEM images of PC/A_S_ composites: (**a**) PC/A_S_10, (**b**) PC/A_S_20, (**c**) PC/A_S_30, (**d**) PC/A_S_40, (**e**) PC/A_S_50, and (**f**) PC/A_S_60. SEM images of PC/A_L_ composites: (**a’**) PC/A_L_10, (**b’**) PC/A_L_20, (**c’**) PC/A_L_30, (**d’**) PC/A_L_40, (**e’**) PC/A_L_50, and (**f’**) PC/A_L_60.

The thermal conductivity (λ) of PC/Al_2_O_3_ composites and their enhancement ratio to pure PC are presented in Figure 1a,b, respectively. Results showed that there was an increment on the λ with an increasing content of Al_2_O_3_. The addition of either larger size or smaller size Al_2_O_3_ particles contributed to an obvious increase of λ when compared with pure PC. However, the enhancement ratio became more appreciable when the filler content reached 40 wt%, regardless of the particle size. This was likely related to the formation of intact thermally conductive pathways at this certain filler concentration [46]. It is worth noting that the λ of PC/A_S_ was higher than that of PC/A_L_ counterparts when the filler concentration was less than 40 wt%; however, an opposite trend was observed when the filler concentration exceeded 40 wt%. The above observation further indicated that the thermally conductive network was likely constructed in the vicinity of 40 wt% Al_2_O_3_, and the difference in the values of λ between both PC/Al_2_O_3_ composites was likely related to the state of filler distribution. For example, a larger number of A_s_ particles were present in PC composites when compared with the A_L_-containing counterparts. According to Li and Shimizu [47], higher shearing conditions were effective for improving the state of distribution of inorganic fillers. Therefore, the improved distribution of spherical particles would be beneficial for improving the λ when the filler content was below 40 wt%. As shown in Figure 2, spherical Al_2_O_3_ particles exhibited a relatively uniform distribution in PC substrate, which was attributed to the high shearing conditions involved during the injection molding process [48]. Moreover, the mean distance between adjacent Al_2_O_3_ particles decreased significantly with increasing filler concentrations, which was critical for improving λ. When the concentration of Al_2_O_3_ reached 40 wt%, samples with larger size particles exhibited more compact filler packing structures and less filler/matrix interfacial thermal resistance [28,49], thereby leading to a higher increment in λ for corresponding PC-based moldings.

Thus, it can be concluded that larger size Al_2_O_3_ particles exhibited a higher efficiency in forming thermally conductive pathways at higher filler concentrations (i.e., >40 wt%), which was likely related to the size effect of the fillers, i.e., Al_2_O_3_ particles. For example, less contact surface was likely formed between adjacent larger size particles when forming a thermally conductive network; the specific surface area of 50 μm Al_2_O_3_ particles was much smaller when compared with their 5 μm counterparts. As such, fewer matrix/filler interfacial defects were generated in PC-based composites with respect to larger size Al_2_O_3_ particles. Under such circumstances, less phonon scattering occurred in terms of 50 μm Al_2_O_3_ particle-containing PC-based composites, which led to a higher λ for corresponding composites when the filler concentration exceeded 40 wt% [49].

### 3.2. Thermal Conductivity of PC/Al_2_O_3_/BN Composites

As reported in previous studies [21,42], planar BN would exhibit a typical orientation along the flow direction due to the intense shearing effect, thereby leading to a higher anisotropy in thermal conduction properties in different directions, i.e., along the flow direction (i.e., in-plane λ) and perpendicular to the flow direction (i.e., through-plane λ). It was also found [42] that the orientation state of planar BN could be altered with the incorporation of spherical Al_2_O_3_ particles, thereby concurrently improving both the in-plane and through-plane λ of injection molded samples. Herein, the influence of the size of Al_2_O_3_ particles on the λ of PC/BN/Al_2_O_3_ composites was highlighted.

**Figure 3:**
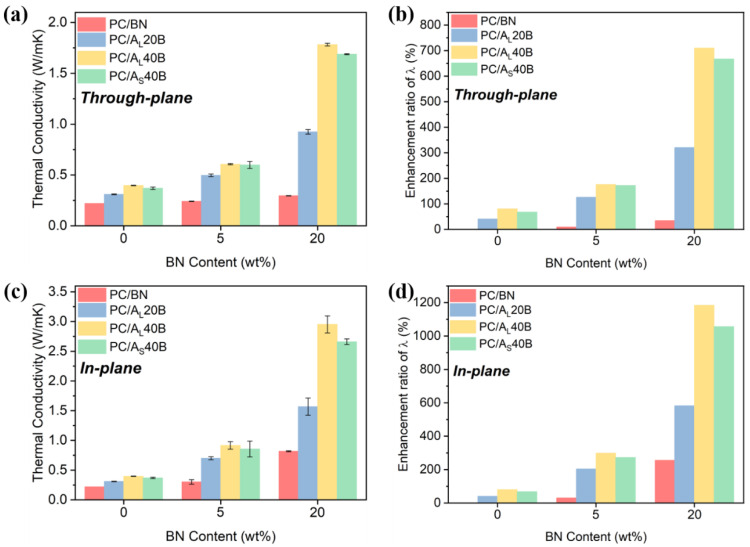
(**a**) Through-plane λ, (**b**) through-plane λ enhancement ratio, (**c**) in-plane λ, and (**d**) in-plane λ enhancement ratio of PC/Al_2_O_3_/BN composites with different Al_2_O_3_ particle sizes and contents.

The λ values of PC/BN/Al_2_O_3_ composites, which were measured along the flow direction and perpendicular to the flow direction, are displayed in Figure 3. Figure 3a,b indicate that no significant enhancement in through-plane λ was observed for PC/BN composites and a great enhancement in in-plane λ when the BN concentration was increased from 5 to 20 wt%, which was related to the preferred orientation and increased addition of planar BN fillers in injection molded samples. Interestingly, there was a significant increase in both the in-plane and through-plane λ with the incorporation of spherical Al_2_O_3_ particles, as shown in Figure 3. For example, the through-plane λ and in-plane λ of PC/A_L_40B20 composites reached as high as 1.78 and 2.95 W/(mK), which were 710% and 1183% higher than those of pure PC, respectively. This was definitely related to the formation of a more compact filler network that contributed to the significant increase of λ. Moreover, the addition of spherical Al_2_O_3_ particles would, to some extent, alter the orientation state of planar BN fillers, thereby contributing to a much greater enhancement in both in-plane and through-plane λ. The influence of particle size of Al_2_O_3_ on the λ of PC/BN/Al_2_O_3_ composites was studied as well. Results showed that samples with larger size Al_2_O_3_ exhibited a much greater increase in λ, which was attributed to the higher efficiency of forming thermally conductive pathways by using larger size spherical particles. In this scenario, the use of A_L_ particles not only reduced the contact thermal resistance between filler/matrix and filler/filler, but they also acted as bridges between adjacent fillers. Both factors contributed to a significant enhancement of λ for subsequent moldings.

**Figure 4:**
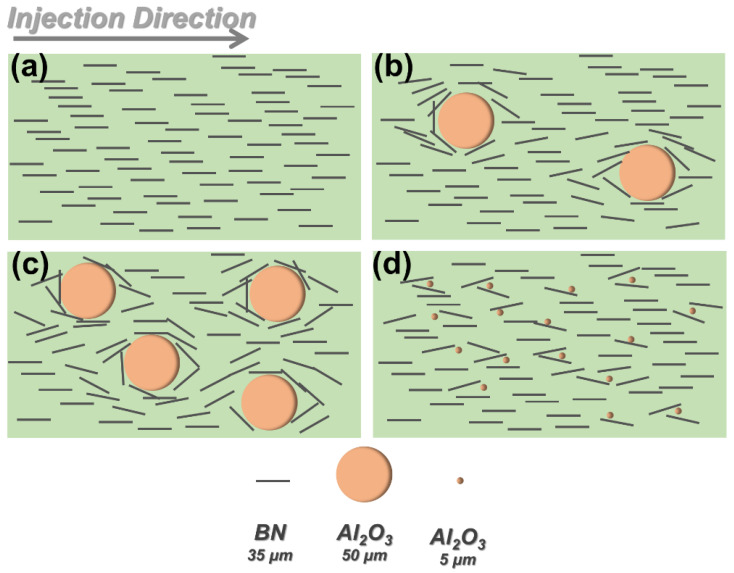
The schematic mechanism for thermal conductivity enhancement of PC/Al_2_O_3_/BN composites with different Al_2_O_3_ particle sizes and contents: (**a**) PC/BN, (**b**) PC/A_L_20B, (**c**) PC/A_L_40B, (**d**) PC/A_S_40B.

The mechanism for thermal conductivity enhancement of subsequent composites is depicted in Figure 4. As shown in Figure 4a, planar BN fillers tended to preferentially align along the melt flow direction due to the predominant shearing effect induced by injection molding. Under such circumstances, injection molded PC/BN composites would exhibit a great discrepancy between in-plane λ and through-plane λ. The preferred orientation of BN was, to some extent, impaired with the incorporation of Al_2_O_3_ particles. As a result, in addition to the presence of Al_2_O_3_ particles, the partial deflection of planar BN would increase the likelihood of constructing thermally conductive pathways across the perpendicular to flow direction, thereby leading to an increase of through-plane λ without much influence on in-plane λ. Moreover, more planar BN fillers would be deflected with the increasing addition of Al_2_O_3_ particles. In this scenario, the difference between in-plane λ and through-plane λ was minimized at higher Al_2_O_3_ loading concentrations. In addition, the size of Al_2_O_3_ particles played a role in determining the deflection state of planar BN. Larger size Al_2_O_3_ particles tended to exert a greater influence on the orientation state of BN fillers, which explained why the PC/BN/Al_2_O_3_ composites with 50 μm Al_2_O_3_ particles demonstrated higher λ than the 5 μm Al_2_O_3_-containing counterparts. As a result, the addition of larger size spherical Al_2_O_3_ particles had a more positive effect on improving the in-plane and through-plane λ of injection molded samples.

### 3.3. Morphology of PC/Al_2_O_3_/BN Composites

**Figure 5:**
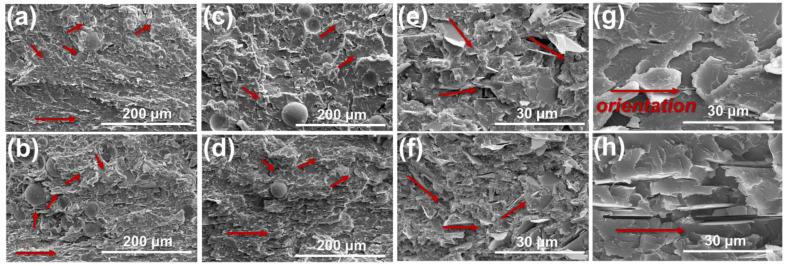
The microstructure of PC-based composites along the flow direction: (**a**) PC/A_L_40B5, (**b**) PC/A_L_40B20, (**c**) PC/A_L_20B5, (**d**) PC/A_L_20B20, (**e**) PC/A_S_40B5, (**f**) PC/A_S_40B20, (**g**) PC/B5; (**h**) PC/B20.

The microstructure of PC/Al_2_O_3_/BN composites is displayed in Figure 5. As shown in Figure 5g,h, planar BN fillers exhibited preferential orientation along the melt flow direction, regardless of filler concentrations. In addition, both BN and Al_2_O_3_ particles showed a relatively uniform distribution within the PC substrate. The presence of BN fillers was labeled using red arrows. Figure 5a–f show that BN fillers, which were located far away from Al_2_O_3_ particles, remained in the preferential orientation state along the flow direction; however, planar BN fillers that were located in the vicinity of Al_2_O_3_ particles showed some deflection due to the steric hindrance effect imposed by Al_2_O_3_ particles. Under such circumstances, the possibility of constructing intact thermally conductive pathways was increased across the thickness direction, i.e., perpendicular to flow direction. As a result, the in-plane and through-plane λ would be concurrently enhanced for injection molded PC/BN/Al_2_O_3_ composites. Moreover, the deflection degree of BN fillers increased with increasing Al_2_O_3_ content and particle size, which is beneficial for building a denser thermally conductive network. Therefore, a larger size Al_2_O_3_ demonstrated an edge over smaller size particles in terms of altering the orientation state of BN fillers, thereby leading to a significant increase of both in-plane and through-plane λ for subsequent PC/BN/Al_2_O_3_ composites. The in-plane and through-plane λ of PC/A_L_40B20 composites reached as high as 2.95 and 1.78 W/(mK), respectively, which were 94.1% and 63.3% higher than PC/A_S_40B20 composites, and 1183% and 710% higher than pure PC, respectively. Thus, incorporating larger size spherical particles was proved effective in improving the λ of planar filler-containing injection molded composites.

### 3.4. XRD Analysis

**Figure 6:**
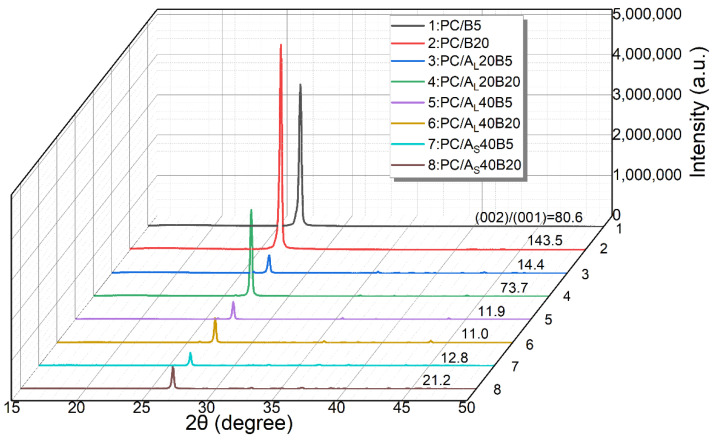
XRD spectra of PC/BN and PC/Al_2_O_3_/BN composites. The influence of particle size and filler content.

**Figure 7:**
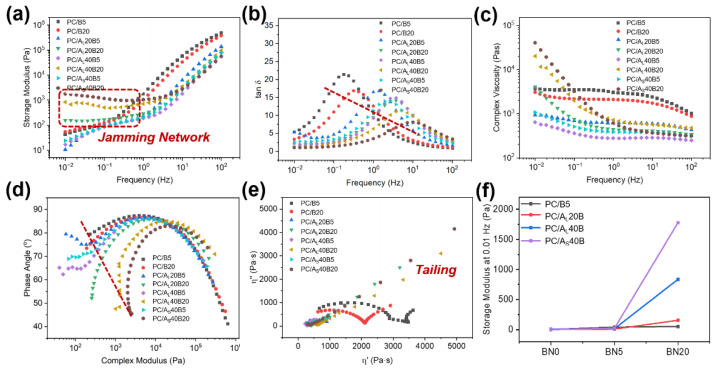
Rheological properties of PC/BN and PC/Al_2_O_3_/BN composites: (**a**) storage modulus vs. frequency, (**b**) tan δ vs. frequency, (**c**) complex viscosity vs. frequency, (**d**) phase angle vs. complex modulus, (**e**) 𝜂″ vs. 𝜂′, and (**f**) storage modulus at 0.01 Hz for different filler-containing composites.

According to literature [50], the orientation degree of BN can be assessed using XRD analysis. Basically, the peak intensity ratio of the (002) plane to the (100) plane of BN was employed to reflect the orientation state of BN [41,51]. The XRD spectra and the peak intensity ratios for related polymer composites are presented in Figure 6. Results showed that the peak intensity ratio of the PC/B20 composite was the highest among the studied systems. The peak intensity ratio of the PC/B20 composite was higher than that of PC/B5, which was related to the reduced melt viscosity related to the incorporation of a larger fraction of planar fillers (see Figure 7c). However, the peak intensity ratios for PC/BN/Al_2_O_3_ composites decreased significantly when compared with binary PC/BN composites, which was ascribed to the filler-induced deflection of planar BN fillers [42].

Specifically, the values of peak intensity ratio decreased with increasing content of Al_2_O_3_, provided that the content of BN and the particle size of Al_2_O_3_ remained the same. In addition, the peak intensity ratios of PC/A_L_/BN composites were higher than those of PC/A_S_/BN counterparts when the concentrations of Al_2_O_3_ particles and BN were the same. Thus, it can be concluded that both increasing the concentration of Al_2_O_3_ particles and using larger size Al_2_O_3_ led to a higher degree of deflection for planar fillers in injection molded samples, which was crucial to improving the through-plane λ of related polymer composites.

### 3.5. Rheological Properties

The viscoelastic properties of PC/BN and PC/Al_2_O_3_/BN composites were studied using a dynamic rheometer, as displayed in Figure 7. The values of storage modulus (G’) as a function of sweeping frequency are given in Figure 7a. Results showed that the values of G’ increased with increasing sweeping frequency and total filler loading concentrations. In particular, the values of G’ at the lowest frequency, i.e., G’_0.01Hz_, increased with total filler concentrations, which was related to the reinforcement effect of the added fillers [52]. A plateau was not visible for PC/BN and PC/Al_2_O_3_/BN 5 wt% composites, suggesting that an intact filler network structure was absent in the above systems. Moreover, a plateau was visible for PC/Al_2_O_3_/BN 20 wt% composites, signifying the existence of an intact filler network that consisted of spherical Al_2_O_3_ and planar BN, which was crucial for improving the λ. Figure 7b,c show that the peak of tan δ shifted to higher frequency regions coupling with a reduction of peak value, and a shear thinning behavior became more noticeable with increasing filler concentrations, which was attributed to the transition from a viscous to solid state of polymer melts arising from the presence of inorganic fillers [36,53].

**Figure 8:**
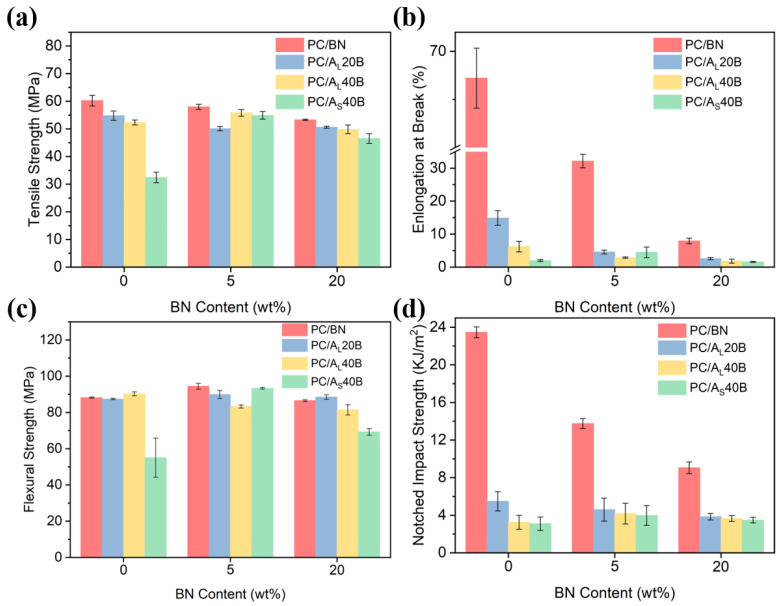
The mechanical properties of PC/BN and PC/Al_2_O_3_/BN composites: (**a**) tensile strength, (**b**) elongation at break, (**c**) flexural strength, and (**d**) notched impact strength.

The microstructural changes of polymer composites were further evaluated using the van Gurp–Palmen and Cole–Cole plots, as given in Figure 7d,e, respectively. Figure 8d showed that the values of phase angle for PC/Al_2_O_3_/BN composites decreased greatly at lower complex modulus regions when the concentration of BN reached 20 wt%. Moreover, the Cole–Cole plot changed from a semicircular to a linear shape with an obvious tailing effect, suggesting that the PC/Al_2_O_3_/BN composites exhibited typical solid-like behavior owing to the formation of three-dimensional thermally conductive pathways [42,54]. Furthermore, Figure 7f shows that there were negligible changes in the values of G’_0.01Hz_ when the filler content of BN was increased from 5 to 20 wt% for binary PC/BN composites, suggesting that no microstructural change (i.e., the formation of a filler network) was detected in PC/BN composites. Moreover, the changes in G’_0.01Hz_ were also insignificant for PC/Al_2_O_3_/BN 5 wt% composites, and such changes became more noticeable when the concentration of BN reached 20 wt%, especially for PC/Al_2_O_3_ 40 wt%/BN 20 wt% composites, which was related to the microstructural change related to the increasing addition of inorganic fillers [52]. It was worth noting that the values of G’_0.01Hz_ at lower frequency regions of the PC/A_S_40B20 composite were higher than its PC/A_L_40B20 counterparts, which suggested that the filler network structure consisting of 5 μm Al_2_O_3_ and BN exerted a much stronger confinement effect on polymer chains, which was likely related to the higher specific surface area of smaller Al_2_O_3_ particles [49]. However, this did not mean that such a filler network structure had a higher efficiency in dissipating heat because the particle size played a positive role in determining the λ of polymer composites [38,49,55].

### 3.6. Mechanical and Electrical Properties

The mechanical properties, including tensile strength, elongation at break, flexural strength, and notched impact strength of PC-based composites are presented in Figure 8. Results showed that the mechanical properties, particularly for elongation at break and notched impact strength, of PC/Al_2_O_3_/BN composites deteriorated significantly with the incorporation of BN and Al_2_O_3_ fillers, which was likely attributed to the poor interfacial interactions between the host substrate and inorganic fillers, as well as the formation of filler network structures that acted as structural defects [16]. Figure 8 shows that the mechanical properties of PC/A_L_40B20 composites were higher than those of PC/A_S_40B20 composites. For example, the tensile strength, elongation at break, flexural strength, and notched impact strength of the former were 49.8 MPa, 1.83%, 81.4 MPa, and 3.66 kJ/m^2^, respectively, whereas those of the latter were 46.5 MPa, 1.59%, 69.2 MPa, and 3.48 kJ/m^2^, respectively. The above results were likely related to the fact that smaller Al_2_O_3_ particles had a tendency to form agglomerates due to their higher specific surface areas and the presence of more filler/matrix interfacial defects [49,56], thereby leading to a much lower mechanical performance for smaller Al_2_O_3_ particle-containing polymer composites. In general, PC/A_L_/BN composites showed reasonable mechanical properties, which demonstrated potential applications in industrial sectors.

**Figure 9:**
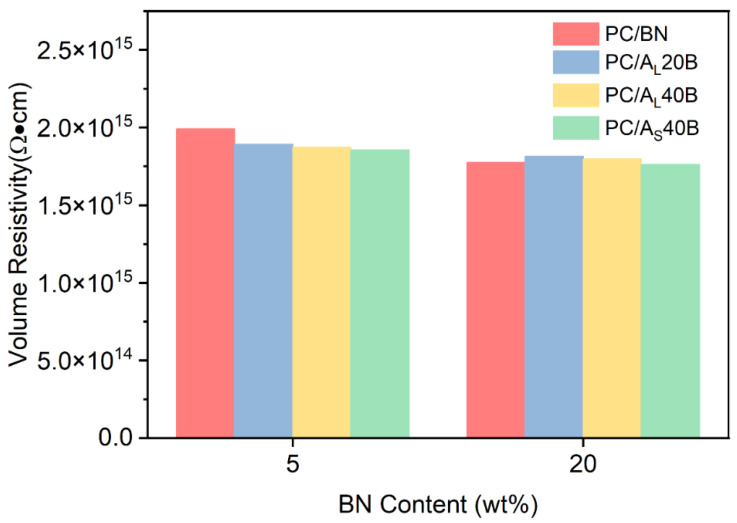
The volume resistance of PC/BN and PC/Al_2_O_3_/BN composites.

The volume resistivity of PC/BN and PC/Al_2_O_3_/BN composites is shown in Figure 9. It can be seen that all samples demonstrated excellent electrical insulation properties, since the volume resistivity of all PC/BN and PC/Al_2_O_3_/BN composites exceeded 10^15^ Ω·cm. Therefore, the prepared polymer composites demonstrated exceptional electrical insulation properties, excellent thermal dissipation properties in both in-plane and through-plane directions of injection molded parts, and reasonable mechanical properties that can be targeted for applications in the fields of electronic devices and battery units, where both thermal conduction and electrical insulation are primary concerns.

## 4. Conclusions

In this work, incorporating spherical alumina (Al_2_O_3_) particles was found to be effective in altering the orientation state of planar boron nitride (BN) fillers in injection molded PC-based composites. Both the in-plane and through-plane thermal conductivity of PC/Al_2_O_3_/BN composites were concurrently enhanced with the addition of spherical Al_2_O_3_ particles. Both increased particle size and loading content of Al_2_O_3_ were found to be crucial in affecting the orientation state of BN fillers, especially for those in the vicinity of Al_2_O_3_ particles, as corroborated by SEM observations and XRD analysis. The deflection state of planar BN fillers and the presence of larger size Al_2_O_3_ particles were beneficial for constructing intact thermally conductive pathways, thereby leading to a significant improvement in through-plane thermal conductivity without much influence on in-plane thermal conductivity. For example, the through-plane and in-plane thermal conductivity of PC/A_L_40B20 composites reached as high as 1.78 and 2.95 W/mK, which were 710% and 1183% higher than those of pure PC, respectively. In addition, the prepared PC/Al_2_O_3_/BN composites demonstrated reasonable mechanical and exceptional electrical insulation properties, which show potential applications in the fields of electronics, battery units, and so forth.

## Figures and Tables

**Table 1:** Formulation of PC/Al_2_O_3_/BN composites.

Samples	PC (wt%)	BN (wt%)	5 μm-Al_2_O_3_ (Small, wt%)	50 μm-Al_2_O_3_ (Large, wt%)
PC/B5	95	5	0	0
PC/B20	80	20	0	0
PC/A_L_10-60	90-40	0	0	10-60
PC/A_S_10-60	90-40	0	10-60	0
PC/A_L_20B5	75	5	0	20
PC/A_L_20B20	60	20	0	20
PC/A_L_40B5	55	5	0	40
PC/A_L_40B20	40	20	0	40
PC/A_S_40B5	55	5	40	0
PC/A_S_40B20	40	20	40	0

## Data Availability

The data presented in this study are available on request from the corresponding author.
